# Molecular Sex Determination in Caenophidian Snakes Using qPCR Amplification of Sex-Linked Genes: Validation and Interspecific Comparison

**DOI:** 10.3390/ani16081175

**Published:** 2026-04-11

**Authors:** George Iulian Enacrachi, Anamaria Ioana Paştiu, Dana Liana Pusta

**Affiliations:** Department of Genetics and Hereditary Diseases, Faculty of Veterinary Medicine, University of Agricultural Sciences and Veterinary Medicine Cluj-Napoca, Calea Mănăştur 3-5, 400372 Cluj-Napoca, Romania; genacrachi@gmail.com (G.I.E.); dana.pusta@usamvcluj.ro (D.L.P.)

**Keywords:** caenophidian snakes, sex determination, GSD, Z-linked genes (*ADARB2*, *ARMC4* and *TANC2*), qPCR

## Abstract

Accurate sex identification in reptiles with genotypic sex determination is essential for breeding management, veterinary care and evolutionary research, yet commonly used methods are often invasive, stressful or unreliable. This study aimed to evaluate a dosage-based quantitative PCR approach for molecular sex determination in caenophidian snakes, using naturally shed epidermal skin as a non-invasive DNA source. Genomic DNA extracted from shed skin was analysed by qPCR targeting conserved Z-linked genes (*ADARB2*, *ARMC4* and *TANC2*), together with autosomal and reference genes, to assess sex-specific differences in gene copy number. Sixteen caenophidian snake species were examined, including taxa for which molecular sexing data are currently scarce or unavailable. The autosomal control gene showed dosage ratios close to parity between sexes, supporting DNA quality and reference gene reliability; meanwhile, Z-linked markers generally exhibited reduced dosage in females relative to males, consistent with a ZZ/ZW sex determination system. These results demonstrate that dosage-based qPCR applied to shed epidermal skin provides a promising and non-invasive framework for molecular sex determination in caenophidian snakes, without compromising animal welfare.

## Simple Summary

Accurate sex identification is a common challenge in veterinary medicine and reptile care because many snake species do not show obvious physical differences between males and females. Traditional sexing methods can be invasive, stressful, or unreliable, especially for young or sensitive animals. This study investigated whether naturally shed snake skin, a material that snakes discard regularly, can be used as a non-invasive and ethical DNA source for determining sex using laboratory analysis. We collected shed skin from several species of advanced snakes and applied a sensitive DNA quantification method (qPCR) to detect differences between males and females. All samples yielded usable DNA, and clear sex-related genetic patterns were identified in most species, allowing accurate sex determination without handling or harming the animals. This non-invasive approach offers important benefits for veterinary practitioners, zoological institutions, snake breeders, and wildlife conservation programs, as it reduces animal stress and risk during routine care and breeding management. By improving animal welfare and supporting responsible practice, our findings contribute to modern veterinary diagnostics and reptile conservation.

## 1. Introduction

Reptiles exhibit a wide diversity of sex-determining mechanisms, and genetic sex determination (GSD) is common across many lineages. Under GSD, sex is specified by sex chromosomes operating under either male heterogamety (XX/XY) or female heterogamety (ZZ/ZW), although the degree of sex chromosome differentiation varies substantially among taxa [1] and in certain species sex determination system turnovers are common events [2]. In reptiles, highly differentiated (heteromorphic) sex chromosomes can coexist with weakly differentiated or homomorphic systems, which complicates both biological interpretation and practical sex diagnosis [3,4]. This variability is particularly relevant for snakes, where caenophidian lineages predominantly exhibit ZZ/ZW with female heterogamety, yet Z/W differentiation may range from clear to cytogenetically cryptic [5,6]. Because molecular assays rely on sex-linked variation that may differ among lineages, methodological validation across species remains essential [7].

Accurate sex identification is important for breeding management, veterinary practice, and research, but traditional sexing methods in reptiles are often limited by age, weak sexual dimorphism, and risks associated with handling. Common approaches include assessment of reproductive behaviour and secondary sexual traits, as well as routine husbandry techniques such as cloacal probing in snakes and related procedures aimed at detecting hemipenial structures. These methods are practical and inexpensive, but they may be unreliable in juveniles or venomous species and they can cause stress or injury if applied incorrectly [8]. Imaging and endoscopic approaches (e.g., ultrasonography, radiography with contrast, computed tomography, and cloacoscopy) can increase diagnostic accuracy, yet they require specialised equipment and trained personnel and may still involve restraint or anaesthesia, limiting their routine use and scalability [9,10,11]. Surgical approaches, including exploratory coeliotomy or laparoscopy and biopsy followed by histology, can provide definitive information but are invasive and impractical for large-scale sexing [10].

Genetic methods provide an alternative framework for sex identification, particularly in lineages where external traits are ambiguous. Cytogenetic and molecular cytogenetic techniques have been instrumental for detecting sex chromosomes and describing their evolution, but classical karyotyping can be technically challenging in snakes because chromosomes are difficult to prepare and often exhibit similar morphology, which reduces the accuracy of purely cytogenetic sex diagnosis [6]. Molecular genetic approaches therefore increasingly facilitate reptile sexing. Conventional PCR assays can identify sex using sex-specific sequences (e.g., W- or Y-linked repeats), but these markers often show narrow taxonomic transferability, as rapidly evolving repeats and non-coding regions may not be conserved even among related species [12]. In caenophidian snakes, multiplex PCR assays have been used successfully across multiple species, illustrating the value of PCR-based sexing while also highlighting that performance may differ among evolutionary lineages [13].

Quantitative PCR (qPCR) extends molecular sexing by enabling relative quantification of gene copy number differences between sexes. In ZZ/ZW systems, females are expected to carry a single copy of Z-linked genes that are absent (or substantially diverged) on the W chromosome, producing a characteristic reduction in female dosage compared with males [6,7]. The usefulness of dosage-based qPCR depends not only on marker choice but also on the quality and practicality of DNA sampling. Invasive sources such as blood or tissue biopsies typically yield high-quality DNA but require animal handling and may be unsuitable for venomous, protected, or stress-sensitive species. Minimally invasive sampling (e.g., buccal swabs) reduces trauma but still requires restraint. Other sources can include tissues obtained post-mortem in deceased individuals, but these are opportunistic and not applicable to routine breeding management. Naturally shed epidermal skin represents a fully non-invasive and widely accessible substrate, particularly in captive collections. Although keratinised material can yield variable DNA quantities [14], it has been shown to provide DNA suitable for downstream genotyping and sequencing workflows in reptiles [15] and offers clear welfare and safety advantages when invasive sampling is undesirable.

By comparing dosage patterns between known males and females and assessing marker performance across taxa, this study evaluates the reliability of shed-skin-based qPCR as a practical diagnostic tool for molecular sex determination in caenophidian snakes and contributes comparative reference data relevant to ongoing questions regarding lineage-specific variation in sex chromosome differentiation [7].

## 2. Materials and Methods

### 2.1. Sample Collection

From 2024 to 2025, a total of 32 samples were collected from three families, six genera and sixteen snake species within the infraorder Caenophidia, as follows: *Pantherophis guttatus*, *Lampropeltis californiae*, *Lampropeltis getula*, *Lampropeltis triangulum hondurensis*, *Boiga cyanea*, *Boiga melanota*, *Boiga multicincta*, *Boiga divergens*, *Boiga lamfasciata*, *Oreocryptophis pulchra*, *Bitis rhinoceros*, *Naja naja*, *Naja kaouthia*, *Naja haje*, *Naja samarensis*, and *Hemachatus haemachatus* (Table 1).

All samples were obtained from naturally shed epidermal tissue collected within 24 h of ecdysis to minimize environmental contamination. Shed skins were handled using sterile gloves and forceps which were previously disinfected with 70% ethanol, and then the samples were individually placed in sealed zip-lock bags together with a labeled data sheet specifying species, sex, age, and date of collection. Following collection, samples were stored frozen until shipment. Transport was arranged by private breeders in accordance with standardized handling and shipping instructions provided by the authors, and was performed within a maximum of 72 h post-collection via courier service. Upon arrival, exuvium samples were stored individually at −20 °C in individualized sealed zip-lock bags until processing.

For all shed skins collected, DNA was extracted from the dorsal trunk region, defined as the mid body area located dorsally between the head and the cloaca of the exuvium, to ensure methodological consistency across samples.

This study included both common and rare captive-bred caenophidian snake species for which sexually confirmed male–female pairs were available. The sex of donor individuals had been previously determined by experienced breeders using cloacal probing. Pair status was additionally based on breeder-reported husbandry records and observations indicating that the two individuals had been maintained together and had a history of mating in captivity. Most individuals were part of established reproductive pairs maintained under captive breeding conditions. The inclusion of individuals with known sex allowed reliable validation of the molecular sexing protocol.

Specimens were originated from licensed private breeders within the European herpetocultural community. Each contributor provided informed consent authorizing the scientific use of the biological material. All procedures complied with institutional and European ethical standards for reptile research. No live animals were handled, subjected to invasive procedures, or euthanized during this study. The study protocol was approved by the Ethics Committee of the University of Agricultural Sciences and Veterinary Medicine of Cluj-Napoca (protocol no. 370/07.04.2023), in accordance with national Law 43/2014 and EU Directive 2010/63/EU.

**Figure 1:**
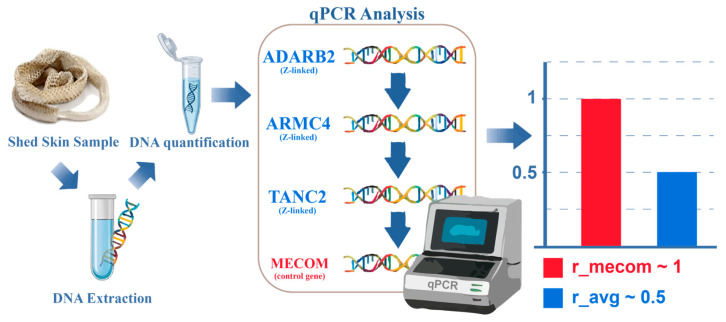
Schematic overview of the experimental workflow and dosage-based qPCR used for molecular sex determination in caenophidian snakes. The diagram illustrates the use of shed epidermal skin as a DNA source, followed by qPCR amplification of reference genes (*EF1A_1* and *EF1A_2*), an autosomal control gene (*MECOM*), and Z-linked genes (*ADARB2*, *ARMC4* and *TANC2*); r_mecom (red) representing the relative dosage ratio for this gene and expected to be close to 1. The parameter r_avg (blue) represents the mean relative gene dosage ratio calculated across all analyzed Z-linked genes and is used for sex determination based on expected differences between males and females.

In this study, we apply and validate a qPCR-based dosage approach targeting conserved Z-linked genes across a phylogenetically diverse set of 16 caenophidian snake species using DNA extracted exclusively from shed epidermal skin, following established dosage principles for reptile sex chromosomes (Figure 1).

### 2.2. DNA Extraction

**Figure 2:**
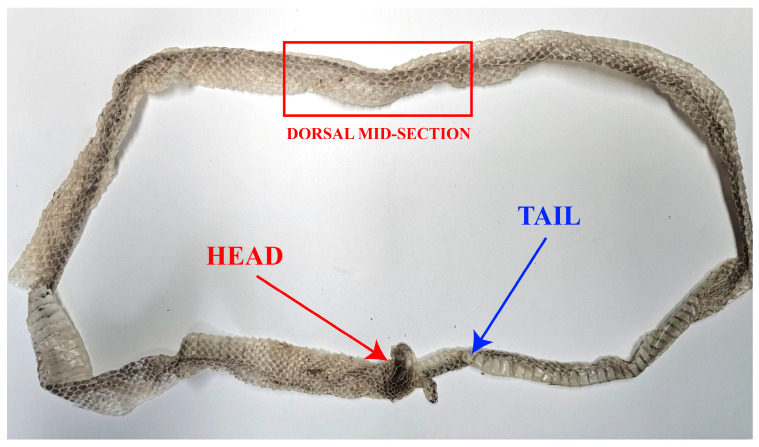
Anatomical localization of the dorsal trunk sampling region. The red rectangle highlights the dorsal trunk region of the exuvium used for DNA sampling. The red arrow indicates the head region, and the blue arrow indicates the tail region. The mid-body dorsal area was consistently selected to ensure methodological standardization across all samples, while ventral scales were deliberately excluded from sampling.

Genomic DNA was extracted from 32 shed skin samples, with subsamples consistently excised from the dorsal trunk region of the exuvium (Figure 2) to standardize sampling across individuals, using the Maxwell^®^ RSC Instrument (Promega, Madison, WI, USA) and the Maxwell^®^ RSC Genomic DNA Kit (Promega, USA), following the manufacturer’s standard protocol with minor modifications.

The shed skin was handled with sterile forceps, cut into small fragments using a sterile surgical blade, and transferred into 1.5 mL Eppendorf tubes for DNA extraction. Each sample (25 mg) was incubated overnight at 56 °C in 300 µL of lysis buffer (LE2) and 30 µL of proteinase K. After incubation, 300 µL of additional lysis buffer was added, followed by brief vortexing and centrifugation at 15,000 rpm for 2 min. The supernatant was transferred to well #1 of the Maxwell^®^ RSC cartridge. Fifteen microliters of RNase A solution were added to well #3, and an elution tube containing 100 µL of elution buffer was positioned in the instrument tray. Automated extraction was then performed according to the instrument’s genomic DNA program. The DNA was stored at −20 °C until use.

DNA yield and purity were assessed for all extracted samples using a ND-1000 spectrophotometer (NanoDrop Technologies, Wilmington, DE, USA).

### 2.3. qPCR Amplification

Real-time PCR (qPCR) was used to determine the variation in the copy number of Z-specific genes between sexes (males-ZZ and female-ZW), following the protocol of Rovatsos et al. [7]. The markers included two reference genes for normalization (*EF1A_1* and *EF1A_2*), one autosomal control gene (*MECOM_5*) and four Z-linked genes (*ADARB2_1*, *ARMC4_3*, *TANC2_1* and *TANC2_2*) (Table 2) [7].

Real-time PCR was performed on Bio-Rad CFX96TM Real-Time System (Bio-Rad Laboratories, Hercules, CA, USA). All DNA samples were amplified in triplicate in a total volume of 15 µL, containing 2 ng genomic DNA, 0.3 µL of each primer (10 pmol/µL), and 7.5 µL of SensiFAST SYBR No-ROX Mix (Meridian Bioscience, Newtown, OH, USA). Thermal cycling conditions were: initial denaturation at 95 °C for 3 min, followed by 44 cycles of 95 °C for 15 s, 56 °C for 30 s, and 72 °C for 30 s. The melting curve program began with initial denaturation for 15 s at 94 °C, followed by cooling to 65 °C, then the measurements were conducted from 65 °C to 95 °C with fluorescence readings taken every 0.1 °C. Ultrapure water was included as a negative control to monitor potential contamination. Crossing-point (Cp) values were automatically determined by the CFX Maestro software, version 2.3, 2021 (Bio-Rad Laboratories, Hercules, CA, US). For each target gene, the arithmetic mean of the three technical replicates was used to minimize measurement error and ensure data accuracy. All calculations were performed in Microsoft Excel (Microsoft Corporation, Redmond, WA, USA).

Each target gene’s dosage was calculated using the Cp values and was then normalized in accordance with the dosage of the reference genes (*EF1A1* or *EF1A2)* from the same DNA sample. Gene dosage ratios were calculated following previously described formulas [6], as defined below:

$$R=2^{Cp\;EEF1A1/EEF1A2\;}/2^{Cp\;gene}$$

$$r=R^{female}/R^{male}$$

where R represents the target-to-reference gene dosage ratio, Cp denotes the crossing point value obtained by qPCR, and r represents the relative gene dosage ratio between females and males for each analyzed gene. Real-time PCR efficiency was not directly evaluated; however, the assay was performed according to a previously validated protocol [7], and a standard amplification efficiency of 100% (E = 2) was assumed, as the same primer systems were used.

For autosomal genes (*MECOM_5*), r should be around 1.0, and for Z-linked genes (*ADARB2_1*, *ARMC4_3*, *TANC2_1* and *TANC2_2*) r should be around 0.5.

Since some reptilian genes that are typically Z-linked may possess paralogous or partially translocated autosomal copies, particularly in taxa with incompletely differentiated sex chromosomes [16,17], minor discrepancies among r values were anticipated. To account for such variability, the mean relative dosage (r_avg) was computed across all Z-linked genes for each individual.

## 3. Results

Genomic DNA was successfully extracted from all shed epidermal skin samples included in the study. DNA concentration varied substantially among species and individuals, ranging from 14 ng/µL in *Lampropeltis californiae* to 540 ng/µL in *Naja samarensis*. Despite this variability, most samples exhibited A260/A280 ratios exceeding 1.8, indicating acceptable DNA purity for qPCR analyses.

The observed Cp values ranged between 24.56 and 36.95 across all markers and samples. For most markers (*EF1A_1*, *EF1A_2*, *MECOM*, *ADARB2_1*, *ARMC4_3*, *TANC2_1*), Cp values were consistently within a typical range. The *TANC2_2* marker exhibited higher Cp values, ranging from 30.58 to 36.95 suggesting lower expression levels; however, amplification remained consistent across replicates.

**Figure 3:**
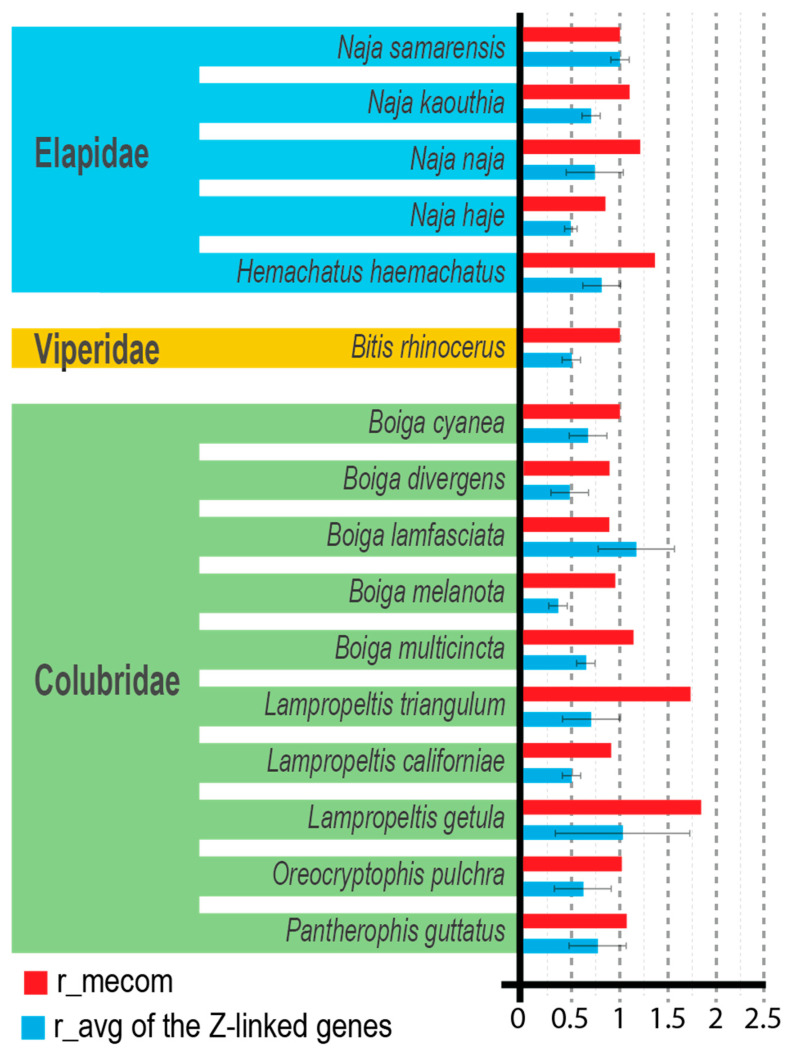
Relative gene dosage ratios (r) between females and males across 16 caenophidian snake species. Red bars (r_mecom) represent the autosomal control gene (*MECOM_5*), while blue bars represent the mean relative dosage (r_avg) of Z-linked genes (*ADARB2_1*, *ARMC4_3*, *TANC2_1* and *TANC2_2*). Error bars represent standard deviation calculated from technical qPCR replicates (Figure adapted from [14]).

Real-time PCR amplification was successful across all analyzed caenophidian snake species. At least one of reference genes (*EF1A1_1* or *EF1A1_2*), the autosomal control gene (*MECOM_5*), and all pairs of primers specific to Z-linked genes were successfully amplified in each species (Figure 3).

The Z-linked genes *ARMC4_3*, *TANC2_1*, and *TANC2_2* were amplified in all tested species, whereas *ADARB2_1* failed to amplify in *Lampropeltis triangulum hondurensis* and *Boiga lamfasciata* (Table S1—Caenophidian snake species analyzed in this study and corresponding qPCR Cp values for all target genes).

Amplification of the autosomal control gene (*MECOM_5*) yielded dosage ratios (r_mecom) ranging from 0.85 to 1.84, with a mean value of 1.11 ± 0.29 (SD) when normalized against at least one reference gene. Two samples with values exceeding 1.5 were observed in *Lampropeltis triangulum* and *Lampropeltis getula*.

For the majority of species, Z-linked markers displayed clear sex-associated dosage differences, with females showing reduced gene dosage relative to males. Mean female-to-male dosage ratios (r_avg) typically ranged from 0.36 to 0.74. Species including *Boiga cyanea*, *B. divergens*, *B. melanota*, *B. multicincta*, *Naja kaouthia*, *N. naja*, *N. haje*, *Bitis rhinoceros*, *Lampropeltis triangulum hondurensis*, *L. californiae*, and *Oreocryptophis pulchra* showed consistent dosage patterns across all Z-linked markers, and molecular sex assignments matched the known sex of reference individuals.

Reduced differentiation between female and male Z-linked gene dosage, with mean ratios approaching parity, was observed in *Naja samarensis*, *Lampropeltis getula brooksi*, and *Boiga lamfasciata*.

## 4. Discussion

The present study demonstrates that naturally shed epidermal skin represents a promising and efficient non-invasive source of genomic DNA for qPCR-based analyses in caenophidian snakes. Although shed epidermal skin is not the optimal source in terms of DNA quantity and purity when compared to DNA obtained via invasive sampling procedures such as blood or tissue collection [13], its practical and ethical advantages nevertheless make it a suitable and often preferred material for molecular sex determination. Even if DNA concentration varied among species and individuals, all samples yielded amplifiable DNA suitable for quantitative assays. The absence of amplification failures supports the suitability of shed skin for molecular sex determination, even when samples originate from different species, breeders, and storage conditions. These findings are consistent with previous reports indicating that keratinised material, despite being traditionally considered challenging, can provide DNA of sufficient quality for molecular applications when appropriate extraction protocols are used [15].

Across all analyzed species, qPCR amplification of reference, autosomal, and Z-linked markers was successful and produced stable dosage patterns. The autosomal control gene (*MECOM_5*) consistently yielded dosage ratios close to parity between sexes, supporting both DNA quality and the validity of the normalization strategy. In most taxa, Z-linked genes showed the expected reduction in female dosage relative to males, consistent with a conserved ZZ/ZW sex determination system characteristic of advanced snakes. This overall pattern supports the applicability of dosage-based qPCR and also supports the use of this approach as a practical tool for molecular sex determination across phylogenetically diverse caenophidian lineages. Overall, these findings are in line with previous studies demonstrating that conserved Z-linked markers yield consistent dosage differences between sexes and can be reliably applied for molecular sex determination across multiple reptile lineages [7].

The majority of species tested in this study exhibited female-to-male dosage ratios well below 1, a pattern consistent with conserved Z-linked and W chromosome degeneration. Specifically, 13 of 16 species (81.25%) showed mean r_avg values < 1. The lowest r_avg was observed in *Boiga melanota* (0.36), followed by *Boiga divergens* (0.48), whereas the highest values still below 1 were recorded in *Haemachatus haemachatus* (0.81) and *Naja naja* (0.74). Such values indicate clear differentiation between Z and W chromosomes and are in agreement with previous genomic and cytogenetic studies reporting long-term conservation of Z-linked regions in snakes [1,6]. The consistency of dosage patterns across multiple Z-linked genes further supports the reliability of these markers for cross-species sex determination in caenophidian snakes.

In contrast, a subset of species analyzed in the present study displayed average dosage ratios approaching parity. Specifically, 3 of 16 species (18.75%) exhibited r_avg values close to or exceeding 1, specifically *Naja samarensis* (1.00), *Lampropeltis getula brooksi* (1.03), and *Boiga lamfasciata* (1.17). Such patterns have been predicted in lineages in which sex chromosomes are weakly differentiated or retain homologous regions shared between the Z and W chromosomes, a condition consistent with partial recombination or incomplete degeneration of the heterogametic chromosome [7]. Recent genomic analyses indicate that, although sex chromosome evolution in snakes has involved multiple transitions and lineage-specific variation, caenophidian species are generally characterized by well-differentiated and evolutionarily stable ZZ/ZW systems. These taxa typically exhibit clear molecular signatures of Z–W divergence, which has enabled reliable genetic sex determination across diverse species. In this context, the occurrence of dosage ratios approaching parity in a small subset of species in the present dataset could reflect lineage-specific variation in marker performance or methodological factors rather than true absence of chromosomal differentiation [18]. For species in which dosage ratios approached a value of 1, further methodological refinement may be required. One potential approach involves the identification of alternative Z-linked markers exhibiting stronger differentiation between the Z and W chromosomes. Additionally, primer redesign targeting different regions of the same genes may improve amplification specificity and reduce the impact of sequence divergence among taxa. Variation in DNA quality inherent to shed epidermal samples may also contribute to minor variability in qPCR-based dosage estimates.

Future studies integrating genomic approaches with molecular validation will be essential to improve the reliability of sex determination across diverse caenophidian lineages. Such observations should therefore be interpreted cautiously and do not contradict the prevailing view of strongly differentiated sex chromosomes in caenophidian snakes, but instead highlight the importance of marker validation across taxa when applying cross-species molecular assays.

Methodologically, this study extends previous dosage-based sexing approaches [7] by averaging dosage values across multiple Z-linked genes rather than relying on individual genes. This strategy reduces the influence of locus-specific irregularities and improves the robustness of sex assignment, particularly in non-model taxa with complex evolutionary histories of sex chromosomes. The consistent performance of the autosomal control gene across all species further supports the validity of this normalization approach.

Molecular sex determination has previously been applied to a restricted set of caenophidian snake species, primarily through PCR-based approaches or dosage analyses validated on a limited number of model or commonly studied taxa. Notably, several species included in the present study, such as *Naja kaouthia*, *Pantherophis guttatus*, *Lampropeltis* spp., *Naja naja*, *Naja haje* and *Boiga cyanea*, have also been sexed in previous studies [7,19]. In this context, at least to our knowledge, the present study expands the taxonomic scope of molecular sex determination in snakes by applying a qPCR framework to additional, previously understudied caenophidian taxa, including representatives of the genera *Boiga*, *Bitis*, *Oreocryptophis* and *Hemachatus*. By extending molecular sexing to these lineages, our results provide new reference data for caenophidian snakes and support the broader applicability of conserved Z-linked markers for cross-species sex determination. Although the study design was optimized for methodological validation rather than population-level assessments, the results clearly demonstrate that shed-skin-based qPCR assays can produce stable and interpretable signals across a wide range of caenophidian snake lineages.

Several limitations must nevertheless be acknowledged. Sample sizes per species were intentionally limited to known male–female pairs, as the primary aim of the study was methodological validation rather than the assessment of intraspecific variation. Consequently, the results should be interpreted as a proof-of-concept demonstrating the applicability of dosage-based qPCR across multiple caenophidian species. Further studies including larger sample sizes per species will be required to confirm the robustness and generalizability of this approach. Moreover, molecular sex determination remains yet unvalidated for many caenophidian species and is currently not feasible for henophidian snakes, primarily because the organization, differentiation, and evolutionary dynamics of their sex chromosomes remain insufficiently characterized. Addressing these knowledge gaps through integrative genomic and cytogenetic research will be essential before molecular sexing can be reliably extended to these lineages.

In practical terms, the ability to perform reliable molecular sex determination using shed epidermal skin has important implications for herpetoculture, zoological institutions, and conservation programs, where accurate sex identification is essential for effective breeding management, population planning, animal welfare and ethical research in veterinary and zoological contexts [20].

## 5. Conclusions

This study demonstrates that qPCR-based quantification of gene copy number variation in conserved Z-linked markers represents a method with encouraging preliminary performance for molecular sex determination in caenophidian snakes with genotypic sex determination. Across the sixteen analyzed species, dosage ratios obtained from four Z-linked genes correctly reflected the known sex of most reference individuals, confirming the applicability of this approach to both common and rare taxa.

The results further show that naturally shed skin proved highly effective, offering an ethical, non-invasive and practical sampling strategy suitable for venomous, protected, or sensitive species and also for juveniles. DNA extracted from shed skin provided sufficient quality and quantity for qPCR amplification of both autosomal and Z-linked genes.

For several of the taxa examined in the present study, *Oreocryptophis pulchra*, *Boiga* spp., *Bitis rhinoceros* and *Hemachatus haemachatus*, we are not aware of previously published molecular sex determination using qPCR data. The present results therefore add new reference information for these species and contribute to extending the taxonomic scope of dosage-based sex determination in caenophidian snakes.

All Z-linked genes included in this study (*ADARB2*, *ARMC4* and *TANC2*) were successfully amplified across the analysed species and contributed to correct sex determination. Averaging dosage values across multiple Z-specific genes reduced the influence of locus-specific irregularities and improved the robustness of sex determination, even in species showing reduced differentiation between the Z and the W chromosomes.

In conclusion, this approach has the potential to enable accurate sex identification without invasive sampling, using shed skin, reduces animal stress, and facilitates broad taxonomic sampling, including venomous or protected species. Taken together, the present results support the use of dosage-based qPCR as an effective first-line tool for molecular sex determination in certain species of caenophidian snakes and provide a foundation for future comparative studies of sex chromosome evolution.

Finally, the present results provide a proof-of-concept supporting the use of dosage-based qPCR for molecular sex determination in caenophidian snakes using shed skin, although further research is needed. Future studies should include larger sample sizes, additional snake species and complementary approaches to refine dosage-based sexing methods and improve our understanding of lineage-specific variation in sex chromosome differentiation.

## Figures and Tables

**Table 1:** Samples collection from each caenophidian snake species.

Family	Species	Number & Sex
*Elapidae*	*Naja samarensis*	1 F
		1 M
	*Naja kaouthia*	1 F
		1 M
	*Naja naja*	1 F
		1 M
	*Naja haje*	1 F
		1 M
*Viperidae*	*Bitis rhinocerus*	1 F
		1 M
*Colubridae*	*Boiga cyanea*	1 F
		1 M
	*Boiga divergens*	1 F
		1 M
	*Boiga lamfasciata*	1 F
		1 M
	*Boiga melanota*	1 F
		1 M
	*Boiga multicincta*	1 F
		1 M
	*Lampropeltis triangulum hondurensis*	1 F
		1 M
	*Lampropeltis californiae*	1 F
		1 M
	*Lampropeltis getula brooksi*	1 F
		1 M
	*Oreocryptophis pulchra*	1 F
		1 M
	*Pantherophis guttatus*	1 F
		1 M

**Table 2:** Primer sequences and target genes used for qPCR-based molecular sex determination in caenophidian snakes, adapted from Rovatsos et al. [7].

Gene	Reason to Amplify	Primer Name	Forward Primer	Reverse Primer	Amplicon Size
*EEF1A1*	normalization	*EF1A_1*	CCTTATTGTTGCTGCTGGTGTT	GTGCTAACTTCTTTGACGATTTCC	189
		*EF1A_2*	TGTGCTGTCCTTATTGTTGCTG	ATGTGCTAACTTCTTTGACGATTTC	199
*MECOM*	autosomal control	*MECOM_5*	AGGAGATTTTGTGAGGGCAAGA	GCTGTTGGAAAGGTAAGACCAG	182
*ADARB2*	Z-linked gene	*ADARB2_1*	CTGCTGGGAATGCGACTGG	GCCTTTCGGAGACTGTGGAG	175
*ARMC4*	Z-linked gene	*ARMC4_3*	AAGCCGTTGCTCCTTTGG	ACCTTTACCACTCCATTTTCGTG	136
*TANC2*	Z-linked gene	*TANC2_1*	ACTGGAGGTGGACTAAAACGG	GGTTCCCATCAGGTCTTTGACT	179
		*TANC2_2*	CCGAAAGGGAGACAGGAACTAC	TGGCAAAATGGACAACACAACC	161

## Data Availability

All the results of the study are presented in the Manuscript.

## Supplementary Materials

The following supporting information can be downloaded at: https://www.mdpi.com/article/10.3390/ani16081175/s1, Table S1: Caenophidian snake species analyzed in this study and corresponding qPCR Cp values for all target genes.

## Abbreviations

The following abbreviations are used in this manuscript:

DNA	Deoxyribonucleic acid
PCR	Polymerase Chain Reaction
qPCR	Quantitative Polymerase Chain Reaction
GSD	Genetic Sexual Determinism
R	Target-to-reference Gene Dosage Ratio
r	Relative Gene Dosage Ratio
r_avg	Mean Relative Gene Dosage
Cp	Crossing point
r_mecom	Relative Dosage ratio for MECOM gene
SD	Standard deviation
